# Additional treatment with mistletoe extracts for patients with breast cancer compared to conventional cancer therapy alone – efficacy and safety, costs and cost-effectiveness, patients and social aspects, and ethical assessment

**DOI:** 10.3205/000312

**Published:** 2022-07-14

**Authors:** Petra Schnell-Inderst, Caroline Steigenberger, Marcel Mertz, Ilvie Otto, Magdalena Flatscher-Thöni, Uwe Siebert

**Affiliations:** 1 Institute of Public Health, Medical Decision Making and Health Technology Assessment, Department of Public Health, Health Services Research and Health Technology Assessment, UMIT TIROL – University for Health Sciences, Medical Informatics and Technology, Hall i. T., Austria; 2 Institute for Ethics, History and Philosophy of Medicine, Hannover Medical School, Hannover, Germany; 3 Institute for Technology Assessment and Department of Radiology, Massachusetts General Hospital, Harvard Medical School, Boston, USA; 4 Center for Health Decision Science, Department of Health Policy and Management, Harvard T.H. Chan School of Public Health, Boston, USA

**Keywords:** mistletoe, biomedical technology assessment, breast neoplasms, health communication, ethics

## Abstract

**Background:**

Chemotherapy is often used in the treatment of breast cancer in women. Side effects such as diarrhea, fatigue, hair loss, fever or disturbances in blood formation impair the women’s quality of life. An essential treatment goal of the accompanying mistletoe therapy (MT) used in complementary medicine is to improve the health-related quality of life during cancer therapy.

**Aim and methods:**

The HTA report on which this article is based examines the medical efficacy and safety, costs and cost-effectiveness, patient and social aspects, and ethical aspects of MT in women with breast cancer. Systematic reviews were conducted for this purpose. The search period of the literature search ranged from 2004 to October 2020.

**Results:**

A total of 2 evidence-based medical guidelines, 3 randomized trials assessing efficacy and 1 additional non-randomized intervention trial, as well as 3 observational studies assessing safety, a cost analysis, 12 cross-sectional studies on patient aspects and 17 articles on ethical evaluation were included. Improvements in health-related quality of life compared to the control group were small to moderate. Due to the high risk of bias in the studies, it is possible that the difference is not caused by MT. One study with a small sample size showed no effect on progression-free survival after 5 years. Studies on the effect of MT on overall survival are lacking. In seven studies, local skin reactions of low and moderate severity were reported in a median of 25% (range 5 to 94%) of patients, and mild to moderate systemic reactions in a median of 2% (range 0 to 8%) of patients. A comparative cost analysis from Germany reported significantly lower medical costs within 5 years after surgery for patients with MT than for patients without MT, but the underlying observational study did not control for systematic bias. With regard to patient aspects, the frequency of use and the reasons for use from the patient’s or practitioner’s point of view were mainly investigated. A median of 25% (range 7 to 46%) of patients with breast cancer and 29% (range 29 to 79%) of treatment providers use MT. The main motivations of patients for use were to reduce side effects, strengthen the immune system and take an active role in the treatment process. Patients felt insufficiently advised. Studies on other aspects are lacking. The ethical evaluation was able to identify 6 overarching themes; the central challenge is the insufficient evidence on efficacy and safety.

## Zusammenfassung

**Hintergrund:**

Bei der Brustkrebsbehandlung von Frauen kommt häufig die Chemotherapie zum Einsatz. Nebenwirkungen wie Durchfall, Müdigkeit, Haarausfall, Fieber oder Störungen der Blutbildung beeinträchtigen die Lebensqualität der Frauen. Ein wesentliches Behandlungsziel der in der Komplementärmedizin eingesetzten begleitenden Misteltherapie (MT) ist es, die gesundheitsbezogene Lebensqualität während einer Krebstherapie zu verbessern.

**Ziel und Methoden:**

Der diesem Artikel zugrundeliegende HTA-Bericht untersucht die medizinische Wirksamkeit und Sicherheit, Kosten und Kosteneffektivität, die Patienten- und sozialen Aspekte sowie die ethischen Aspekte einer MT bei Frauen mit Brustkrebs. Hierzu wurden systematische Übersichtsarbeiten durchgeführt. Der Recherchezeitraum der Literatursuche reichte von 2004 bis Oktober 2020.

**Ergebnisse:**

Insgesamt wurden 2 evidenzbasierte medizinische Leitlinien, 3 randomisierte Studien zur Bewertung der Wirksamkeit und zusätzlich 1 nichtrandomisierte Interventionsstudie sowie 3 Beobachtungsstudien zur Bewertung der Sicherheit, eine Kostenanalyse, 12 Querschnittstudien zu Patientenaspekten und 17 Fachartikel zur ethischen Bewertung eingeschlossen. Verbesserungen der gesundheitsbezogenen Lebensqualität gegenüber der Kontrollgruppe waren gering bis moderat. Aufgrund des hohen Verzerrungsrisikos in den Studien ist es möglich, dass der Unterschied nicht durch die MT bedingt ist. Eine Studie mit kleiner Fallzahl zeigte nach 5 Jahren keinen Effekt auf das progressionsfreie Überleben. Studien zum Effekt der MT auf das Gesamtüberleben fehlen. In sieben Studien zeigten sich als Nebenwirkungen lokale Hautreaktionen geringen und mittleren Grads im Median bei 25% (Spannweite 5 bis 94%) der Patientinnen und leichte bis mittlere systemische Reaktionen im Median bei 2% (Spannweite 0 bis 8%) der Patientinnen. Eine vergleichende Kostenanalyse aus Deutschland berichtete wesentlich geringere Krankheitskosten innerhalb von 5 Jahren nach der Operation für Patientinnen mit MT als bei Patientinnen ohne MT, jedoch erfolgte in der zugrundeliegenden Beobachtungsstudie keinerlei Kontrolle für systematische Verzerrungen. Hinsichtlich Patientenaspekten wurde vor allem die Häufigkeit der Anwendung und die Gründe für die Anwendung aus Patientinnen- oder Behandelndensicht untersucht. Im Median wenden 25% (Spannweite 7 bis 46%) der Patientinnen mit Brustkrebs und 29% (Spannweite 29 bis 79%) der Behandelnden eine MT an. Hauptmotivation zur Nutzung waren die Reduktion von Nebenwirkungen, die Stärkung des Immunsystems, und eine aktive Rolle im Behandlungsprozess einzunehmen. Patientinnen fühlten sich ungenügend beraten. Zu weiteren Aspekten fehlen Studien. Die ethische Bewertung konnte 6 übergreifende Themen identifizieren; zentrale Herausforderung ist die unzureichende Evidenzlage zu Wirksamkeit und Sicherheit.

## Background

Cancer is the second most common cause of death in Germany after cardiovascular disease [1]. For women, breast cancer ranks first in terms of cancer-specific deaths and new cancer cases, accounting for 18,736 deaths and 30.8% of all new cancer cases in 2016 [1], [2]. The age-standardized incidence of breast cancer in 2016 was 112.2 women per 100,000, and the age-standardized incidence of death was 23.4 women with breast cancer per 100,000 [2]. The mean (median) age of onset is 64 years, and the relative survival rate after ten years is 82%, indicating that the chances of cure and survival are relatively good [2]. In addition to surgery and depending on the tumor status, chemotherapy can be used to treat breast cancer. Chemotherapy is usually associated with side effects such as diarrhea, fatigue, hair loss, fever, and blood formation disorders. This significantly impairs the patients’ quality of life during treatment [3], [4].

Mistletoe preparations are used for the concomitant treatment of cancer in Germany. A major goal of treatment is to improve the health-related quality of life during therapy, which is limited due to the toxicity of chemotherapy [3]. Mistletoe therapy is classified as complementary or integrative medicine [5]. The biochemical mechanisms of action of mistletoe preparations are attributed to mistletoe lectins and viscotoxins, which have a non-specific immunostimulant and a cytostatic effect [6], [7]. Anthroposophical mistletoe extracts without standardized lectin content and mistletoe preparations registered as herbal remedies with standardized lectin content are sold on the German market [8]. All mistletoe preparations are available without prescription. The costs of mistletoe therapy as part of palliative treatment for patients with metastatic breast cancer are covered by the statutory health insurance. Mistletoe therapy as a part of adjuvant treatment for non-metastatic breast cancer is not covered due to the uncertain evidence currently available. The HTA report by Lange-Lindberg et al. [9] investigated mistletoe therapy as an adjuvant treatment to reduce toxicity of chemotherapy of malignant diseases. It concluded that only in the case of breast cancer were there indications that an adjuvant mistletoe therapy could improve the patient’s quality of life. The current German medical guideline on early detection, diagnosis, and therapy of breast cancer states that mistletoe therapy does not improve the survival of patients with breast cancer, and an improvement in quality of life is questionable due to insufficient data [4]. This raises the question of whether there is new evidence on medical efficacy and safety that can clarify these questions. In addition, the present article aims to systematically examine costs and cost-effectiveness, patients’ aspects and social implications, and ethical questions associated with concomitant mistletoe therapy.

## Research questions

1. The following research questions were investigated to evaluate medical efficacy and safety: Does the administration of mistletoe preparations reduce patient-relevant side effects of conventional chemotherapy and improve health-related quality of life in patients with breast cancer compared to chemotherapy without concomitant therapy with mistletoe preparations? Does the administration of mistletoe preparations in addition to conventional chemotherapy influence progression-free survival or overall survival in patients with breast cancer?
2. To evaluate economic aspects, the following research questions were investigated: What are the differences in the costs of concomitant mistletoe therapy and what is the cost-effectiveness of concomitant mistletoe therapy compared to treatment without mistletoe preparations?
3. The evaluation of patients and social aspects examined the following research questions: What are the attitudes, experiences, perceptions, and knowledge of patients and professionals regarding concomitant mistletoe therapy? Are there barriers to accessing mistletoe therapy for interested patients? What does communication about concomitant mistletoe therapy look like, and are there particular points that should be communicated to ensure adequate uptake?
4. The ethical evaluation examined the following research questions: Which ethical aspects on an individual, social, and professional level are relevant for mistletoe therapy as a concomitant therapy in patients with non-metastatic or metastatic breast cancer compared to conventional therapy alone? What is the outcome of an evaluation and weighing of the identified ethical aspects and challenges regarding the use of mistletoe therapy as concomitant therapy in patients with breast cancer, i.e., under what conditions is the use of concomitant mistletoe therapy in adjuvant and palliative treatment of breast cancer ethically justifiable?

## Evaluation of efficacy and safety

### Methods

#### Main inclusion and exclusion criteria for primary studies, systematic reviews, HTA reports, and evidence-based guidelines

Published randomized controlled trials (RCT) on the efficacy and safety of adjuvant or palliative mistletoe therapy in patients with breast cancer were included that examined outcomes on at least one of the following outcome measures: adverse effects of standard therapy, health-related quality of life, overall survival, progression-free survival, adverse effects of mistletoe therapy. For the assessment of adverse effects of mistletoe therapy, observational studies were also included. In addition, systematic reviews and HTA reports with literature searches from 2004 onwards that met the above inclusion criteria as well as evidence-based clinical practice guidelines for the treatment of breast cancer with statements on complementary medicine from 2008 onwards were included.

#### Literature search, selection, assessment of study quality, data extraction, and evidence synthesis

A systematic literature search was conducted in the following databases: MEDLINE, EMBASE, Cochrane Library, and HTA Database of the Centre of Reviews and Dissemination. The search period ranged from January 1^st^, 2004 to March/April 2017. Between April 2017 and October 2020, further publications were identified through a continuous update function in PubMed. Search terms for the disease (breast cancer) and for the therapy (mistletoe preparations) were combined in the form of free text and database-specific thesaurus terms. An Internet search was conducted using the Grey Matters checklist of the Canadian Agency for Drugs and Technologies in Health [10]. Guidelines were also searched for in three guideline databases. All references were selected independently by two authors using the predefined inclusion and exclusion criteria. Differences were resolved through discussion. In addition, a hand search was conducted in the bibliographies of the identified included systematic reviews, HTA reports, primary studies, and guidelines.

Cochrane’s Risk of Bias tool [11] was used to assess potential bias of the RCT, and the *A*ppraisal of *G*uidelines for *RE*search & *E*valuation (AGREE) tool [12] was used to assess the methodological quality of the guidelines.

Identified HTA reports and systematic reviews were used to search for additional primary studies. From the included studies and guidelines, the relevant characteristics and results were extracted by one person and checked for accuracy by a second. The results from the primary studies on the efficacy and safety of concomitant mistletoe therapy in breast cancer were presented in evidence tables and figures and described in summary texts. Due to the heterogeneity of the studies, no meta-analyses were performed. The recommendations of the clinical guidelines on concomitant mistletoe therapy are described in text form.

### Results

In the literature search, 242 references were identified after removing duplicates. After screening of titles and abstracts, 80 articles were reviewed in full text and a total of seven studies [13], [14], [15], [16], [17], [18], [19], [20], [21], [22], [23], [24], [25] were included in the information synthesis for the evaluation of clinical efficacy and safety. For the description of guideline recommendations, two guidelines [4], [26] were included after reviewing 199 references and 163 full texts.

#### Efficacy

No study on the effect of concomitant mistletoe therapy on overall survival was identified. A three-arm RCT [24], [25] with a small sample size comparing two different mistletoe preparations with a placebo control showed no effect of concomitant mistletoe therapy on progression-free survival after five years: 72.4 and 67.9% with concomitant therapy and with the mistletoe preparations, 78.6% in the control group (p-value compared to the control group 0.551 and 0.746, respectively). Three RCT [19], [20], [21], [22] (719 patients) investigated the change in health-specific quality of life and adverse symptoms of the standard therapy after 15 and 18 weeks. Four different validated quality of life instruments (FACT-G [Functional Assessment of Cancer Therapy – General] [27], [28], [29], GLQ-8 [Global Life Quality] [30], [31], Spitzer analog scale [31], [32], EORTC QLQ-C30 [European Organisation for Research and Treatment of Cancer] [33], [34], [35], [36]) were used. In one study [20] (352 patients), the FACT-G questionnaire was used. The group difference of the total score (range 0 to 80 points, control group score in placebo group at baseline 50 points), after 15 weeks was four points (p-value <0.0001) in favor of the mistletoe group. A value for a minimal clinically relevant difference is not given. Two studies [19], [20] (598 patients) used the GLQ-8 symptom scale, a visual analog scale measured in mm. In one study [19] (272 patients), the change in the GLQ-8 symptom scale (range 0 to 800 mm) after 15 weeks was 30 mm (p-value 0.0121) in favor of the mistletoe group. The values at baseline ranged from 128.9 to 171.5 mm [19] in four study arms. In the second study [20] (352 patients), the difference in favor of the mistletoe group was 40 mm (p-value <0.01). At baseline, the value was 150 mm. The indication of a minimal clinically relevant difference was missing. However, the difference was considered clinically relevant by the study authors. The Spitzer analog scale questionnaire on quality of life (theoretically possible range 0 to 100) showed a change in favor of the mistletoe group after 15 weeks in the same two studies. The difference was 5.8 and approximately 5.0 mm, respectively. The values at baseline varied between 29 and 46.4 mm [20] in the study arms. Again, there is no statement about a clinically relevant minimum difference. One study [21], [22] (95 patients) investigated two mistletoe preparations in parallel versus a control group and used the EORTC QLQ-C30 questionnaire. The EORTC QLQ-C30 consists of five functional scales, one total health subscale and nine symptom scales, which are reported individually (range 0 to 100 points each). A difference of at least five points is considered clinically relevant [33], [34], [35], [36]. After 18 weeks, both groups with mistletoe preparations had statistically significant values (p-value <0.05) above five points difference from the control group in three functional scales (role, social, and emotional) and five symptom scales (nausea and vomiting, insomnia, loss of appetite, diarrhea, and financial problems). The differences compared to the control group ranged from 6.01 to 14.09 points [21], [22].

#### Risk of bias in studies

All studies were adequately randomized and have low data loss in the analysis. For two studies [19], [20], it is unclear whether group allocation was concealed. One study [21], [22] was unblinded and two studies [19], [20] were double blinded with a placebo. However, due to the frequent local reactions, unblinding is likely. Because health-related quality of life is a patient-reported outcome, there is a high risk of performance and detection bias. The risk of bias due to attrition was low or unclear, and the risk of bias due to other causes was low.

#### Safety

A total of seven studies [13], [14], [15], [16], [17], [18], [19], [20], [21], [22], [23] with 1,951 patients were included for the question of undesirable side effects due to concomitant mistletoe therapy, three [19], [20], [21], [22] of which are the RCT already identified for the question on efficacy. From these seven studies, only the data from the study arm with concomitant mistletoe therapy were taken. Known side effects of mistletoe injections are local skin reactions and systemic reactions such as fever and flu-like symptoms. In six studies with eight intervention groups, a median of 25.2% (range 4.6 to 94%) of the patients had local reactions of low or moderate severity.

Seven studies [13], [14], [15], [16], [17], [18], [19], [20], [21], [22], [23] reported numbers of mild and moderate systemic reactions in the study populations, with a median of 1.95% (range 0 to 8.2%).

### Discussion

In summary, there is no evidence on the effect of concomitant mistletoe therapy on overall survival, and little evidence that concomitant mistletoe therapy has no effect on progression-free survival after five years. The improvements in health-related quality of life compared to the control group are small to moderate. Since there was a high risk of bias in all studies due to the lack of or inability to maintain blinding, it is possible that the difference is not caused by the concomitant mistletoe therapy. It seems unclear whether the study results are fully transferable to the current conditions in German-speaking countries. The included studies were conducted before 2004 or between 2005 and 2007. All studies took place in Eastern or South-Eastern Europe. Recent developments in breast cancer therapy as well as differences in health care and culture in the German-speaking countries compared to these countries could have an influence on the transferability of the study results.

Overall, there is great uncertainty as to whether the presented small to moderate improvements in health-related quality of life or the reduction of symptoms caused by chemotherapy and disease can be causally attributed to mistletoe therapy. Additionally, it is uncertain whether the results of the studies can be transferred to the current conditions in German-speaking countries. Thus, new studies could lead to a change in the overall assessment of the evidence.

New randomized studies on health-related quality of life should try to solve the central methodological problem of maintaining double blinding.

The present review on clinical efficacy and safety is subject to several limitations. The comprehensive systematic literature search was conducted in 2017. However, automatically updated notifications from one of the bibliographic databases and the review of current systematic reviews [37], [38], [39], [40], [41] allowed us to confirm that no additional recent studies (with a censor date of December 2020) are available. Full texts of two RCT [42], [43] identified in previous reviews were not available, so we did not report the results from those studies. These studies had small case numbers ranging from 17 to 46 patients, poor reporting quality, and a high risk of bias. Accordingly, inclusion or exclusion of these studies would not change the outcome of the evaluation. Due to the heterogeneity of the studies, we did not consider it appropriate to conduct a meta-analysis for efficacy or safety outcomes. However, the statistical uncertainty that can be reduced by a meta-analysis was not the main limitation. Our main limitation was the high potential for systematic bias in the effect sizes.

## Evaluation of costs and cost effectiveness

### Methods

#### Main inclusion and exclusion criteria for studies

For the assessment of costs and cost-effectiveness of concomitant mistletoe therapy, we applied the same inclusion and exclusion criteria as for the assessment of efficacy and safety for study population, intervention, and comparator intervention. Outcome measures were the additional costs of concomitant mistletoe therapy, as well as additional costs per life year gained, or quality-adjusted life year. All health economic study types were included. The evaluations had to relate to the German-speaking region.

#### Literature search, selection, assessment of study quality, data extraction, and evidence synthesis

Literature search and selection were conducted analogously to the assessment of clinical efficacy and safety. Standardized extraction forms were used for data extraction, and the criteria catalog for the methodological quality of health economic studies of the German Scientific Working Group Technology Assessment in Health Care [44] was used for quality assessment. Study characteristics and results were summarized in evidence tables.

### Results

In the literature search, 243 references were identified after removing duplicates. After screening of titles and abstracts, five full-text articles [45], [46], [47], [48], [49] were reviewed, and one study [45] was included in the information synthesis.

A comparative cost analysis of concomitant mistletoe therapy was identified based on 2005 prices and with therapy data from between 1990 and 2000. The underlying multi-center, retrospective cohort study [50] investigated the efficacy and safety of concomitant mistletoe therapy in patients with breast cancer during routine follow-up in 53 randomly selected hospitals and medical practices in Germany. Direct medical costs in inpatient and outpatient settings as well as indirect costs for a loss of productivity of up to 90 days were collected after surgery and the completion of adjuvant chemotherapy or radiotherapy. In total, the data of 741 patients were analyzed – 167 patients with concomitant mistletoe therapy, 514 patients with standard therapy without concomitant mistletoe therapy, 60 patients who had switched between standard therapy alone and concomitant mistletoe therapy [45].

The average total costs within five years for a patient with concomitant mistletoe therapy were 4,504 euros, compared to 9,996 euros for a patient with standard therapy. The difference was mainly due to inpatient costs and lost productivity costs. Data on statistical uncertainty were not reported. The study has many limitations in data quality, but the main weakness is that the observational study [50] on which the economic evaluation is based did not control for systematic bias with the study design.

### Discussion

Studies on the cost-effectiveness of concomitant mistletoe therapy were not found.

The large cost difference in favor of concomitant mistletoe therapy in the comparative cost analysis cannot be attributed to a causal effect of mistletoe therapy due to the high risk of bias, as the data are based on an observational study [50] without any control for systematic bias due to confounders. However, data on the level of education as well as other prognostic factors that are known confounders were not collected in the study. It therefore remains unclear whether an accompanying mistletoe therapy can reduce the costs of illness in the follow-up care of breast cancer.

As long as the question of the clinical effectiveness of concomitant mistletoe therapy is uncertain, the question of cost-effectiveness cannot be answered.

## Evaluation of patients and social aspects

### Methods

#### Main inclusion and exclusion criteria for studies

All published studies on mistletoe therapy in patients with breast cancer that refer to the German-speaking region and have reported results on relevant outcome measures on patients and social aspects were included. Relevant outcome measures are the following: use of mistletoe therapy, knowledge, attitude, acceptance, satisfaction, experiences, expectations of users of mistletoe therapy, access to mistletoe therapy, type and extent of communication and information on mistletoe therapy, and mistletoe therapy evaluation by patients. The outcome measures could be reported from the perspective of the patients, from the perspective of their caregivers or family members, or from the perspective of the treating health professionals. Quantitative and qualitative study types without further restriction could be included.

#### Literature search, selection, assessment of study quality, data extraction, and evidence synthesis

For the literature search, in addition to the cross-domain search and the comprehensive search for gray literature described above, a systematic database search was conducted in twelve databases from the fields of medicine, economics, sociology, and psychology. All references were selected independently by two authors using the predefined inclusion and exclusion criteria. Differences were resolved through discussion. In addition, a hand search was conducted in the bibliographies of the included primary studies. The checklists of the Critical Appraisal Skills Program [51], [52] were used in original or adapted form to assess the risk of bias. Relevant characteristics and results were extracted from the included studies by one person and checked for accuracy by a second. Results for quantitative primary studies were summarized and presented in evidence tables. The qualitative studies were analyzed using qualitative content analysis.

### Results

The literature search identified 318 references after removing duplicates. After screening of titles and abstracts, 96 articles were reviewed in full text. A total of twelve cross-sectional studies [53], [54], [55], [56], [57], [58], [59], [60], [61], [62], [63], [64] – nine [53], [54], [55], [56], [59], [61], [62], [63], [64] on the patient perspective, three [57], [58], [60] on the practitioner perspective – and three qualitative studies [65], [66], [67] were included in the information synthesis for the assessment of patient aspects and social aspects.

Ten studies [53], [54], [55], [56], [59], [61], [62], [63], [64], [66] with 3,421 patients with breast cancer report results on the frequency of use of complimentary and integrative medicine (CIM) and mistletoe therapy. A median of 24.9% (range 7.3 to 46.3%) of patients with breast cancer use mistletoe therapy. Seven [53], [54], [55], [62], [63], [64], [66] of the ten studies with 2,482 patients report sociodemographic, disease-related, or therapy-related characteristics that are more frequent in patients with breast cancer when using complementary methods than in patients not using them. CAM users (CAM = complementary and alternative medicine) are younger, have a higher level of education, and a more severe disease course.

Three studies [57], [58], [60] with 654 professionals (mostly physicians) report results on the frequency of use of CIM and concomitant mistletoe therapy and on characteristics of the professionals who use them. A median of 29.3% (range: 29.3 to 79.2%) use concomitant mistletoe therapy. CIM and concomitant mistletoe therapy users of the treating professionals are typically older and work in private practice or have a higher hierarchical status in the hospital.

Seven studies [56], [59], [57], [58], [60], [65], [66] describe the respondents’ attitudes towards concomitant mistletoe therapy and what benefits they expect from its use, including two cross-sectional studies [56], [59] with 197 patients with breast cancer, three cross-sectional studies [57], [58], [60] with 654 treating professionals, and two qualitative studies [65], [66] with 43 patients with breast cancer. In one study [56], trust in the counselor and perceived competence regarding CIM was important for 90% of CIM users because it made them feel that the use of CIM was good for them. In another study [59], patients want to leave nothing untried (47%), want an active role in treatment (47%), want to complement conventional therapy (31%), want to have a gentle treatment free of side effects (18%), or did not respond to conventional therapy and mistletoe therapy (3.6%) [59]. Specifically, the use of mistletoe therapy is associated with the expectation of having fewer side effects with conventional cancer therapy and stimulating the immune system [56], [59], [66].

Three studies [57], [58], [60] investigated the reasons given by health professionals for or against the use of concomitant mistletoe therapy. With few exceptions, only the study by Kalder et al. [57] also provides quantitative data. Reasons for the application of mistletoe therapy among healthcare professionals were the patients’ wish (82%), the patients’ motivation (62%), the expansion of their own range of services (59%), their own conviction (55% [57] and 66% [60]) or the belief in the ineffectiveness of conventional therapy (46%). Reasons against using them include that time is lost (32.9%), unconventional methods are too expensive (30.4%) and the use of conventional methods is discouraged (27.3%). In addition, specific expertise and personnel are lacking [58].

An online cross-sectional study [56] with 80 patients and two qualitative studies with [65], [66] 34 patients report results on the assessment of patient information and doctor-patient communication. Seventy of the patients thought that the consultation time on CIM was not sufficient and only 53% thought that the doctor was well informed about CIM [56]. The qualitative studies showed that patients would like personal advice on CIM or mistletoe therapy from their attending physician, and not only advice but also not mentioning CIM or mistletoe therapy can be interpreted as advice.

No study reported access restrictions. As a particular challenge in the application of mistletoe therapy, only physicians in the hospital setting reported in one study that CIM is not part of routine care and reimbursement schemes, which makes its application hardly possible and billable [58].

#### Risk of bias and methodological quality of studies

The results of the cross-sectional studies are considered valid. It is questionable whether the recruitment of the samples in the individual study centers was suitable to obtain representative results for all patients with breast cancer in Germany. The respective study setting and the self-selection of the participants could have influenced the results. The transferability of the results from the studies to the target population of the HTA report this article is based on is thus unclear, and is reinforced by deficiencies in data quality, such as a non-transparent description of patient characteristics in an anonymous online survey. The qualitative studies [65], [66], [67] show different methodological deficiencies. For example, the relationship between respondents and interviewers was not adequately considered or described in any of the studies, and the data analysis was not reported sufficiently in many places.

Overall, the evidence base for all outcomes from the patient’s perspective, except for the frequency of utilization, is low, with 197 patients in cross-sectional studies and 43 in qualitative studies. The evidence base on the perspective of the treating professionals is somewhat better, with three studies [57], [58], [60] and 654 participants. However, two [57], [60] of the three studies are already 20 years old.

### Discussion

Approximately one quarter of patients with breast cancer make use of concomitant mistletoe therapy. In the setting of the identified cross-sectional studies from the treating perspective, about 29% of healthcare professionals offer concomitant mistletoe therapy to their patients.

Deficits became apparent in knowledge about mistletoe therapy, communication, and patient information. Patients feel insufficiently informed about mistletoe therapy and would like more and longer personal advice from a competent specialist, preferably from an oncologist or general practitioner. The possibility to obtain information independently on the Internet does not replace this need for counseling. This lack of knowledge was also stated by the treating professionals who commented on their own uncertainty regarding mistletoe therapy due to the unclear evidence on its efficacy and the lack of knowledge about complementary methods in general.

Mistletoe therapy or CIM in general should be proactively discussed in doctor-patient consultations. The goal for doctor-patient communication should be to address the need for counseling, determine which need should be or is fulfilled by mistletoe therapy, and address that need in therapy planning.

The S3 Guideline of the Association of the Scientific Medical Societies “Complementary Medicine in the Treatment of Oncological Patients” [68] can provide the basis for evidence-based decisions in doctor-patient communication. When developing, using, and recommending information for patients, the Internet should not be disregarded as a source of information.

There is a lack of high-quality surveys of patients on all patient aspects, except for the use of mistletoe therapy, as well as more recent surveys of treating professionals. Additional high-quality surveys of both patients and healthcare professionals could form the basis for improved patient information and communication processes.

Limitations of the present analysis are that it may not have been possible to identify all relevant studies through the literature search. There is too little data available on knowledge, attitudes, acceptance, satisfaction, experiences, expectations, access, type and scope of communication, information on mistletoe therapy available to patients, and evaluation of this information by patients. Whether the included studies are representative of patients with breast cancer and the treating professionals in Germany is questionable.

## Ethical evaluation

### Methods

The ethical evaluation consisted of three parts. In the first part, a search was conducted to determine whether there are existing recommendations or guidelines for dealing with the ethical aspects of mistletoe therapy in breast cancer. In the second part, a systematic review was conducted with the aim of identifying ethical aspects of mistletoe therapy in breast cancer. For this purpose, the included literature was evaluated using methods of qualitative content analysis. In a third part, the results of the literature review were assigned to the ethical principles relevant to medicine and public health (i.e., benefit, harm, efficiency (costs), justice, self-determination, and legitimacy) using an ethical framework. In addition, further aspects were added based on theoretical reflection.

The following databases were used for the literature search: PubMed, PhilPapers, Sowiport, and Ethicsweb. Ethical aspects were identified using the principles of the framework mentioned above (i.e., something is an ethical aspect if the aspect has a relation to benefit, harm, justice, etc.).

The ethical aspects extracted from the included literature were categorized using the framework with which they were identified; and by means of inductive categorization (i.e., formation of superordinate categories based on comparison of the material found), they were classified into specific subcategories.

### Results

Seventeen professional articles [69], [70], [71], [72], [73], [74], [75], [76], [77], [78], [79], [80], [81], [82], [83], [84], [85] were included. In the evaluation of these articles, 22 ethical aspects were identified. Through the supplementary theoretical reflection, four additional ethical aspects were identified that were not represented in the included literature. Similarly, the literature search did not yield any specific contributions on professional ethical aspects. No further aspects were added from the other domains. This made it possible to identify a total of 26 ethical aspects that could potentially be relevant for concomitant mistletoe therapy in breast cancer.

The 26 ethical aspects were divided into six main categories. These correspond to the ethical principles used (benefit, harm, self-determination, justice, efficiency (cost), and legitimacy). For the principles ‘justice’ and ‘legitimacy’, no ethical aspects could be assigned. For a more concrete thematic classification of the aspects, eight subcategories were also formed. Of the 26 aspects, 21 were classified as ethical risks (risk of insufficient consideration of an ethical principle) and five as ethical challenges (balancing between ethical principles required). These ethical aspects were concretized and combined into eight criteria (what is ethically required?) and four conflicts (what needs to be weighed?).

In summary, six overarching themes can be identified based on the aspects and criteria/conflicts:

General topic(s) that may in principle also apply to other therapies in the field of complementary and integrative medicine or to conventional therapies include:

1. Difficulties in informed consent to therapy (e.g., what content is required to ensure sufficient information, or how to ensure freedom of consent from too much influence, especially from outside).
2. A possible danger of a “therapeutic misunderstanding”, e.g., an erroneous conviction on the part of a patient that a therapy used only palliatively is part of a causal therapy (i.e., serves to treat the breast cancer).
3. Difficulties in weighing the potential harms and benefits of the therapy, including interactions and side effects, or failure to do so.

In addition, there are more specific issues that arise particularly in concomitant mistletoe therapy, including:

1. Problems of quantitatively and/or qualitatively insufficient evidence to assess potential benefits and harms and underlying problems of evidence generation for mistletoe therapy.
2. The possible lack of communication or information between physicians, complementary/integrative medicine practitioners, and/or patients as to whether mistletoe therapy is or has been started and with which modalities.
3. Difficulties in dealing with possible placebo or nocebo effects in mistletoe therapy.

### Discussion

A pivotal point in the ethical evaluation of palliative as well as adjuvant mistletoe therapy for breast cancer is the problematic evidence situation regarding efficacy and safety. On the one hand, some ethical aspects or criteria/conflicts relate directly to this. For example, all aspects that have to do with the assessment and weighing of potential benefits and harms are directly related to efficacy and safety of mistletoe therapy. On the other hand, other ethical aspects or criteria/conflicts are dependent on the evidence situation, such as the conditions for successful informed consent to a therapy (e.g., information about potential benefits and harms, information about the evidence situation or the state of science) or the communication between doctors and complementary/integrative medicine practitioners due to possible discrepancies in the assessment of effectiveness.

In these decisions – at least as long as the potential for harm is considered minimal and financing is unproblematic –, the autonomy of the patients will be ethically decisive to a particular extent in each individual case. Furthermore, in the case of a possible adjuvant (non-palliative) mistletoe therapy, which must be paid out of pocket by the patients, additional questions could arise regarding the fairness of the legal regulation since the statutory health insurance only reimburses mistletoe therapy as a palliative, not as an adjuvant therapy.

Beyond the individual case, decisions must also be made at the political level as to whether reimbursement of palliative mistletoe therapy for breast cancer should continue in view of the uncertainties surrounding its effectiveness. At the institutional level, it is unclear whether this uncertainty can be resolved in the medium and long term through several new and/or better studies.

However, in the case of palliative as well as adjuvant mistletoe therapy for breast cancer, special emphasis must be placed on the danger of both an unfair assessment of the potential benefits and harms and/or insufficiently neutral education about the therapy due to prejudices based on its affiliation with complementary or integrative medicine. In this context, both possible negative and positive prejudices (e.g., due to ideological convictions) must be considered, as an uncritical and overly-optimistic assessment of the benefit potentials and a distorted explanation of the benefits to the patient could be judged as ethically problematic.

## Summary discussion, conclusions, and recommendations

The HTA report on which this article is based contains a systematic literature review on efficacy and safety, costs and cost-effectiveness, patients and social aspects, and ethical evaluation.

Within the scope of the HTA report, no randomized controlled trials on the clinical efficacy of concomitant mistletoe therapy regarding overall survival in patients with breast cancer could be identified. One study [24], [25] with a small sample size showed no difference in disease-free survival after five years between patients with and without concomitant mistletoe therapy.

There is evidence from three randomized controlled trials [19], [20], [21], [22] that side effects of chemotherapy – as measured by symptom scales – are reduced, and health-related quality of life – as measured by functional scales – is increased. However, the effects are rather small to moderate. It is uncertain whether these effects could be due to systematic bias of the only subjectively measurable outcome measures due to insufficient blinding in the studies.

Known side effects of mistletoe therapy, such as mild and moderate local reactions, that were recorded in these three RCT [19], [20], [21], [22] and four other non-randomized studies [13], [14], [15], [16], [17], [18], [23] are common but of low magnitude. Possible interactions between anticancer drugs and mistletoe extracts, which could be due to immune stimulation, were not investigated in the included studies.

There are no sufficiently valid studies on the costs and cost-effectiveness of concomitant mistletoe therapy.

Given this overall uncertain evidence, the extension of the funding of mistletoe therapy to adjuvant therapy by statutory health insurance cannot be recommended. Beyond individual cases, decisions must also be made at the political level as to whether reimbursement of palliative mistletoe therapy for breast cancer should continue in view of the uncertainties surrounding its efficacy.

Although mistletoe therapy is approved and available without prescription, it is hoped that further randomized trials will be conducted to reduce the uncertainty regarding efficacy in improving health-related quality of life and to capture possible interactions between anticancer drugs and mistletoe extracts as possible side effects. Efficacy in terms of overall survival and progression-free survival should also be investigated. A review on the methodological challenges of randomized trials of mistletoe therapy and corresponding approaches to solving them could then be used to develop an adequate study design.

Representative surveys should also be conducted on knowledge, attitudes, acceptance, satisfaction, experiences, expectations, access, type and extent of doctor-patient communication, and information on mistletoe therapy.

The uncertainty of the existing data makes it even more important to advise patients neutrally and competently, regardless of the attitude of the practitioners or counselors themselves towards mistletoe therapy. The few studies from the patient point of view with small numbers of participants indicate that patients often do not feel adequately advised by professionals, and patients do not inform the practitioner about mistletoe therapy if the practitioner’s attitude towards it is negative. An important component of patient counseling should firstly be the comprehensible and transparent communication of the fact that there is currently no scientific proof of whether mistletoe therapy relevantly improves overall survival time or progression-free survival in patients with breast cancer. Secondly, it should be made clear that an improvement in health-related quality of life is to be assessed as rather moderate and cannot be regarded as assured. In addition, it should be pointed out that interactions between cancer drugs and mistletoe therapy could possibly occur due to the immune stimulation, but there is currently insufficient data on this as well. For patients who would like to use medical information from the Internet, references should be made to trustworthy websites that contain information on concomitant mistletoe therapy.

## Notes

### HTA report

This article is the short version of the HTA report of the same title [86].

### Competing interests

The authors declare that they have no competing interests.
